# Adaptation insights from comparative transcriptome analysis of two Opisthopappus species in the Taihang mountains

**DOI:** 10.1186/s12864-022-08703-5

**Published:** 2022-06-24

**Authors:** Ning Chen, Hao Zhang, En Zang, Zhi-Xia Liu, Ya-Fei Lan, Wei-Li Hao, Shan He, Xing Fan, Gen-Lou Sun, Yi-Ling Wang

**Affiliations:** 1 College of Life Science, Shanxi Normal University, Taiyuan, 030031 China; 2 Triticeae Research Institute, Sichuan Agricultural University, Chengdu, 611130 China; 3 Department of Biology, Saint Mary’s University, Halifax, B3H3C3 Canada

**Keywords:** Opisthopappus, Comparative transcriptome, Differentiation, Adaptation

## Abstract

*Opisthopappus* is a major wild source of Asteraceae with resistance to cold and drought. Two species of this genus (*Opisthopappus taihangensis* and *O. longilobus*) have been employed as model systems to address the evolutionary history of perennial herb biomes in the Taihang Mountains of China. However, further studies on the adaptive divergence processes of these two species are currently impeded by the lack of genomic resources. To elucidate the molecular mechanisms involved, a comparative analysis of these two species was conducted. Among the identified transcription factors, the bHLH members were most prevalent, which exhibited significantly different expression levels in the terpenoid metabolic pathway. *O. longilobus* showed higher level of expression than did *O. taihangensis* in terms of terpenes biosynthesis and metabolism, particularly monoterpenoids and diterpenoids. Analyses of the positive selection genes (PSGs) identified from *O. taihangensis* and *O. longilobus* revealed that 1203 genes were related to adaptative divergence, which were under rapid evolution and/or have signs of positive selection. Differential expressions of PSG occurred primarily in the mitochondrial electron transport, starch degradation, secondary metabolism, as well as nucleotide synthesis and S-metabolism pathway processes. Several PSGs were obviously differentially expressed in terpenes biosynthesis that might result in the fragrances divergence between *O. longilobus* and *O. taihangensis*, which would provide insights into adaptation of the two species to different environments that characterized by sub-humid warm temperate and temperate continental monsoon climates. The comparative analysis for these two species in *Opisthopappus* not only revealed how the divergence occurred from molecular perspective, but also provided novel insights into how differential adaptations occurred in Taihang Mountains.

## Introduction

Adaptation is a topic of fundamental interest to researchers in ecology, evolution, and conservation [1–4]. When different populations experience heterogeneous environments, natural selection can drive phenotypic divergence while modulating underlying genomic architectures, which induces populations to adapt to their habitats [3, 5]. Adaptative divergence has been well verified as a major mechanism that initiates evolutionary diversification and speciation [6, 7]. There are myriad variations in the combination of phenotypic traits and environmental factors [8]. Variation created by mutation, the raw material for evolutionary change, is translated into phenotypes by flux through metabolic pathways and by the topography and dynamics of molecular networks [9]. Thus, empirical adaptation tests are of principal importance as they explore the balance between the evolutionary processes that shape populations (e.g., strength of selection in promoting local adaptation) relative to non-adaptive or even maladaptive factors such as gene flow, recombination, mutation, and genetic drift [10–12].

Transcriptome sequencing is a fast and cost-effective approach for characterizing entire RNA data of an organism [13]. It has been employed to identify the functional elements in the genomes, metabolic pathways, and differentially expressed genes (DEGs) of plants [14–21], particularly those that lack a sequenced reference genome, or non-model species [22]. Moreover, comparative transcriptome analysis has become a powerful tool for investigating plant growth, development, and physiology at the transcriptional and metabolic levels [23], which provides opportunities to estimate transcriptome-wide divergence and identify loci under selection [17]. Especially, comparative RNA-sequencing studies between intimately related species can not only provide additional genomic resources but also offer data related to the processes of speciation or adaptive evolution [16, 24].

The *Opisthopappus* Shih genus belongs to the family Asteraceae, which includes two closely related species (*O. taihangensis* Shih and *O. longilobus* (Ling) Shih) [25]. These two species generally grow within the cliff cracks and rock gaps of the Taihang Mountains in China. They have similar morphological characteristics, but can be distinguished based on the style of pinnatisect leaves and the presence or absence of bracteal leaves [26, 27]. For *O. longilobus*, most stems and leaves are pinnatifid with one pair of bracteal leaves; *O. taihangensis* has bipinnatifid stems and leaves with no bracteal leaves [27]. Based on our observations in a common-garden, the stems of *O. longilobus* are more brittle than those of *O. taihangensis*, and *O. longilobus* individuals are relatively smaller than those of *O. taihangensis*. Further, *O. taihangensis* more easily survives than *O. longilobus* when they were transplanted into a common garden. Meanwhile, both *O. taihangensis* and *O. longilobus* can exude unique fragrances. HPLC (High Performance Liquid Chromatography) and FTIR (Fourier Transform Infrared Spectrometery) analyses revealed that the chemical compositions and contents of chlorogenic acid, apigenin, and quercetin were different between the two species (data not published).

During the long-term evolution of *Opisthopappus*, the dramatic geological changes and significant topographical barriers of Taihang Mountains could limit the large-scale population expansion and gradually introduce heterogeneity within different populations of *O. taihangensis* and *O. longilobus* [28]. *O. taihangensis* and *O. longilobus* were likely diverged during the early Miocene under a strengthening East Asian monsoon [29]. Subsequently, the dramatic climatic transformation and complex topography of the Taihang Mountains further promoted differentiations between the two species and their populations [29]. At the present, *O. taihangensis* and *O. longilobus* occupy different niches due to difference in growth habits [30]. According to the current geographical distribution of *O. taihangensis* and *O. longilobus*, we presumed that adaptive divergence processes might have played an additional role in maintaining the differentiation of the two *Opisthopappus* species that inhabit different regions of the Taihang Mountains [29]. Whether driven by morphological, genetic, or metabolic aspects, differentiation proceeded between *O. taihangensis* and *O. longilobus*.Previous genetic and phylogeographic studies on nuclear DNA, plastid DNA and transcriptome data have shown significant variation between the two species [28, 30–34]. However, the molecular mechanisms underlying these phenomena remains unclear, where a paucity of genetic resources has made further studies on the neutral and adaptive divergence processes of *Opisthopappus* is a challenging task.

In this study, transcriptome sequencing and comparative analysis of *O. taihangensis* and *O. longilobus* were conducted to reveal the expression of differentially genes under different selective pressures and the main metabolic pathways patterns in the two species. These analyses will provide useful data regarding their adaptation to specific habitats and enable the exploration of adaptive evolutionary signatures between the two species. It is anticipated that the data generated in this study will enhance our knowledge of the regulatory networks and pathways involved in assessing the roles of evolutionary factors on the adaptation, and speciation of *Opisthopappus*.

## Materials and methods

### Plant materials

*O. taihangensis* distributes mostly across Henan and Shanxi Provinces, which are located along the southern Taihang Mountains and belong to a sub-humid warm temperate climate; while *O. longilobus* generally grows in Hebei and Shanxi Provinces, which reside along the northern Taihang Mountains with a temperate continental monsoon climate.

**Fig. 1:**
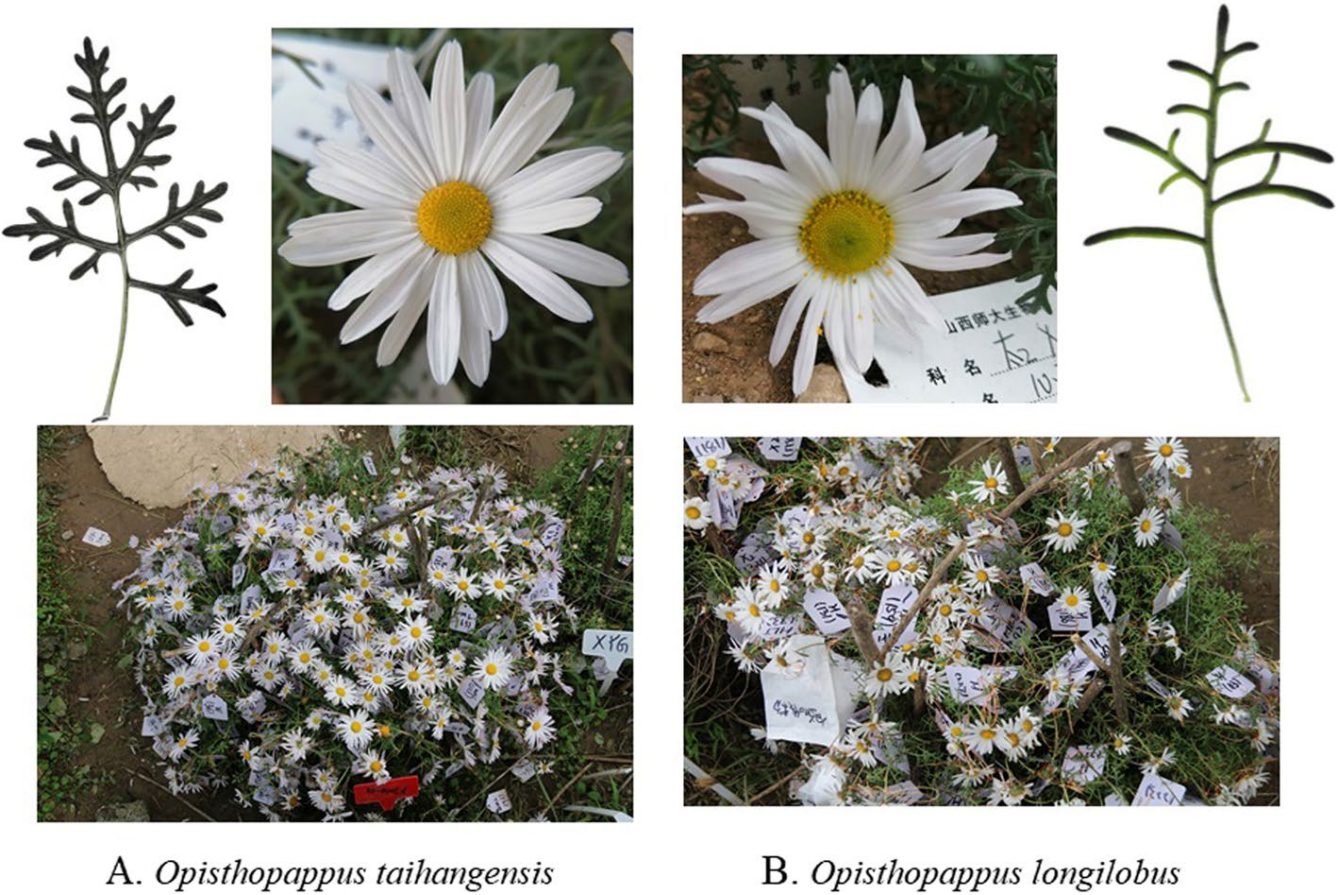
The picture of *Opisthopappus taihangensis* (**A**) and *Opisthopappus longilobus* (**B**)

To elucidate the molecular mechanisms of differentiation between *O. taihangensis* and *O. longilobus*, the perennial plants of the two species (Fig. 1A and B) were sampled from Shanxi Province where the two have adjacent distribution as closely as possible (Study protocol comply with relevant institutional, national, and international guidelines and legislation, and collection of O. taihangensis and O. longilobus have obtained the permission from the College of Life Sciences of Shanxi Normal University). During the same growth period of *O. taihangensis* and *O. longilobus* at July in 2015, fresh mature leaves from the same branch and position of each species were sampled. Each individual from the same population was collected from different locations at least 10 m apart. Then mixed leaves of five individuals were collected as a sample. Three samples (biological replicates) were tested for each population, including taihangensis1, taihangensis2 and taihangensis3 for *O. taihangensis* and longilobus1, longilobus2 and longilobus3 for *O. longilobus*. The samples were frozen in liquid nitrogen (immediately stored at -80 °C) and then sent to Shanghai Personal Biotechnology Co., Ltd. The data were generated in accordance with a transcriptome analysis program in our previous studies [28].

### TF identification and expression analysis

PlantTFDB (http://planttfdb.cbi.pku.edu.cn/index.php) is a plant transcription factor database that includes the sequences of 58 plant transcription factor families from 165 plant species [35]. Clean reads were assembled de novo using Trinity software, and the sequences of unigenes were Blastx aligned to the transcription factor database PlantTFDB, where the best of these with E values of less than 1e-5, were screened as the annotation data of the unigenes. Candidates that contained DNA binding domains were recognized by GO annotation for final TF identification.

An in-depth transcriptome analysis would facilitate an improved perception of the species under study [15, 21, 36]. An advanced bioinformatics tool (MapMan) was used to analyze and compare transcriptional responses between phylogenetically species, and especially useful at the level of functional categories in cross-species comparisons [37]. This analysis allows us to explore gene categories from large data sets to extract meaningful information. In this study, MapMan [38, 39] was employed to comprehensively interpret transcriptome data and visualize the functionalities of associated genes. Through the MapMan, it was evident that significant variations between two species were conspicuous with respect to differentially expressed genes [40]. At the same time, we employed metabolic overview installed in the MapMan tool to identify the primary and secondary metabolic pathways related to the genes associated with transcription factors (TFs) [41]. MapMan metabolism encompasses photosynthesis pathways, carbohydrate metabolism, N-dependent pathways (e.g., amino acid metabolism), as well as cell wall, lipid, and secondary metabolism. Meanwhile, the differentially expressed TFs and transporters were isolated by Fisher’s Exact Test (*P*-value < 0.05) and enrichment fold ≥ 1.5 compared with the whole genome background.

### PSG identification and expression analysis

#### Orthologous contigs and estimation of substitution rates

The assembled sequences were initially employed to predict the ORF (Open Read Frame) regions. Subsequently, the ORFs were blasted using OrthoMCL (http:// orthomcl.org/orthomcl/) software. Orthologous pairs with identities of < 60% were excluded, and only 1:1 orthologous pair in both lineages were retained. Next, based on the BLAST results, Markov clustering was used with the Markov Cluster algorithm (MCL) method. Aligned sequences showing a 90% identity were defined as pairs of putative orthologues. The best-hit sequences of each cluster were then used in subsequent analyses.

The KAKS_CALCULATOR software [42, 43] was used to calculate nonsynonymous (dN) and synonymous (dS) substitution rates of the single-copy orthologous genes, and the dN/dS ratios of each putative orthologous pair using the YN [44] algorithm in PAML software. The dN/dS ratio is an estimate of natural selection acting on the genes. The estimated values and its proportion were assigned to one out of five selection scenarios: (i) dN/dS = 0–0.6 (strong negative selection), (ii) dN/dS = 0.61–0.9 (weak negative selection), (iii) dN/dS = 0.91–1.5 (neutral selection), (iv) dN/dS = 1.51–5 (weak positive selection), and (v) dN/dS > 5 (strong positive selection) [45]. Alignments with dS values of < 0.01 and dS or dN > 2 were discarded. Very high dN/dS (> 10) were also removed as they indicated bias.

According to the standard errors computed from YN00 for a Student’s t-test, the statistical significance of the differences between dN and dS was analyzed [44]. The pair of othologous with a *P*-value < 0.05 and dN/dS > 1.5 was designated as preliminary candidate under positive selection. A *P*-value < 0.01 and dN/dS > 5 were used as threshold for candidate genes with stronger positive selection.

#### GO classification analysis

The identified positive selection genes were processed to retrieve associated Gene Ontology (GO) terms describing biological processes, molecular functions, and cellular components using TBtools software. Expression levels of all the positive selection genes in replicates were assessed by mapping high quality (HQ) filtered reads using BOWTIE2 [46]. Mapped reads were further normalized using the Fragments Per Kilobase Per Millions (FPKM) method. The significance of the difference in GO term abundance between the two datasets was tested using the Fisher’s exact test of the GOSSIP package [47] implemented in BLAST2GO [48].

#### Pathway enrichment analysis

To analyze the PSG pathways between the two species, we employed two annotation methods (KEGG and MapMan) to compare the expressional differentiation between *O. taihangensis* and *O. longilobus*. KEGG (http://www.kegg.jp/) [49] pathway annotation was performed using the BLASTX algorithm with E-values < 1.00E^−5^ and enabled ‘sensitive mode’ against the KEGG (Kyoto Encyclopedia of Genes and Genomes) gene peptide database. Subsequently, the corresponding KO identifiers, EC numbers and pathway categories were parsed using customized Perl scripts. For the KEGG function classification, unigenes were annotated into five categories of KEGG metabolic pathways (cellular processes, environmental information processing, organismal systems, metabolic and genetic information processing).

For MapMan analysis [41], transcripts expressed in both species were annotated with the TAIR database (Arabidopsis homologs). A MapMan BIN file, with a hierarchical ontology system for basil genes, was prepared using Mercator [50, 51], by comparing transcripts against already-classified proteins. BIN level information and gene identifiers were also derived from the same database.

## Results

### Transcription factors(TFs)

Numerous studies have shown that transcription factors (TFs) are involved in the physiological processes and adaptation of plants, which can regulate gene expression during all developmental and growth stages. Of the 483 unigenes, 406 were transcription factors belonging to 41 transcription factors families, including bHLH, bZIP, etc. Among them, the bHLH family was the most abundant with 67 transcription factors, whereas the second most abundant family was FAR1 with 50 transcription factors, followed by the NAC (40 transcription factors), M-type (24 transcription factors), MYB and MYB related (24 transcription factors), and WRKY (22 transcription factors) families (Table 1).

**Table 1:** Transcription factors (TFs) identified in this study

TFs	Unigene counts	TFs	Unigene counts	TFs	Unigene counts	TFs	Unigene counts
bHLH	67	G2-like	10	C2H2	4	HD-ZIP	2
FAR1	50	Trihelix	10	CO-like	4	LBD	2
NAC	40	bZIP	10	Dof	4	STAT	2
M-type	24	MYB	9	S1Fa-like	4	CAMTA	1
WRKY	22	BES1	8	TCP	4	E2F/DP	1
Nin-like	19	GRAS	8	ERF	3	GATA	1
B3	17	GeBP	7	HB-other	3	HRT-like	1
MYB-related	15	SBP	7	NF-YB	3	NY-YA	1
EIL	13	YABBY	7	NF-YC	3	RAV	1
C3H	11	HSF	5	ARF	2	ZF-HD	1

While, families having 10–19 transcription factors included Nin-like (19 factors), B3 (17 factors), EIL (13 factors), C3H (11 factors), G2-like (10 factors), and Trihelix (10 factors). Other families had less than 10 transcription factors, such as BES1 (8 factors), SBP (7 factors), and TCP (4 factors). Seven families (ZF-HD, RAV, NF-YA, HRT-like, GATA, E2F/DP, and CAMTA) had only one factor. Among the 406 TFs, 309 were shared by *O. taihangensis* and *O. longilobus*, while 46 were exclusive to *O. longilobus* and 13 were exclusive to *O. taihangensis*.

**Fig. 2:**
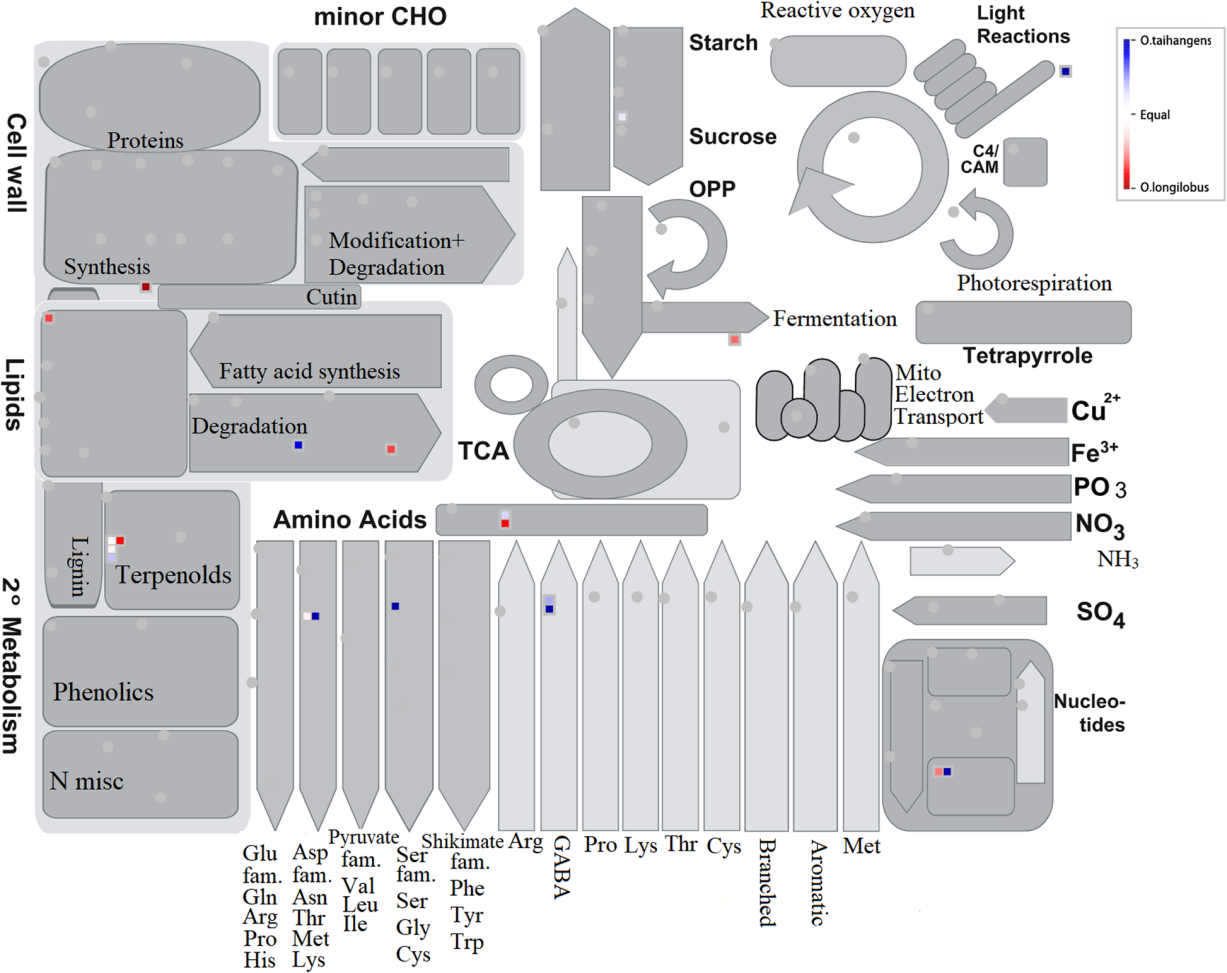
An overview of the metabolic pathways of transcription factors (TFs). Each inset presents a differentially expressed gene. Red or blue indicate *O. taihangensis* and *O. longilobus*, respectively

To systematically assign functions to the 406 TF genes, we employed the diverse overview tools installed in MapMan. The metabolism overview assigned the 406 TF genes as follow: seven for amino acid metabolism, four for secondary metabolism, three for lipid metabolism, one for cell wall metabolism, one for major carbohydrate metabolism, one for photosystems, and one for fermentation (Fig. 2).

Significant expression differentiation between *O. taihangensis* and *O. longilobus* occurred in the terpenoid metabolic pathway, in which the transcription factors of *O. longilobus* were obviously more active than those of *O. taihangensis*. Furthermore, for the amino acid metabolism pathways, the TFs were observed to be more numerously expressed in *O. taihangensis*, whereas in other pathways the TFs were almost equally expressed between the two species (Fig. 7A).

### Positive selection genes(PSGs)

Positive selection genes (PSGs) are those under natural selection, which reflect the great adaptation of species to factors that are intimately linked to their survival under specific environmental conditions. Thus, PSGs were analyzed to explore the variations in the development and adaptation of the two *Opisthopappus* species.

Based on the predicted ORF, we identified 59,753 pairs of putative orthologous contigs between *O. taihangensis* and *O. longilobus* using ORTHOMCL. The one-to-one, reciprocal best method for elucidating orthologous proteins generated 50,587 putative orthologous pairs. After filtering the gene pairs annotated with different proteins in the Swiss-Prot database, 38,986 pairs of putative orthologs were finally identified and used in subsequent analyses. Of these, 11,598 pairs had only either synonymous or non-synonymous substitutions, and 38,986 pairs had both types of substitutions, for which the dN/dS ratios were calculated. Among the 38,986 pairs of putative orthologs, 1596 putative othologous pairs had dN/dS ratios = 0.91–1.5, which indicated ongoing neutral evolution. And 36,187 with dN/dS ratios between 0.0001 and 0.9 suffered negative selection that might have experienced relaxed purifying selection and/or unfixed mutations without altering the encoded amino acid sequence during the speciation period.

**Fig. 3:**
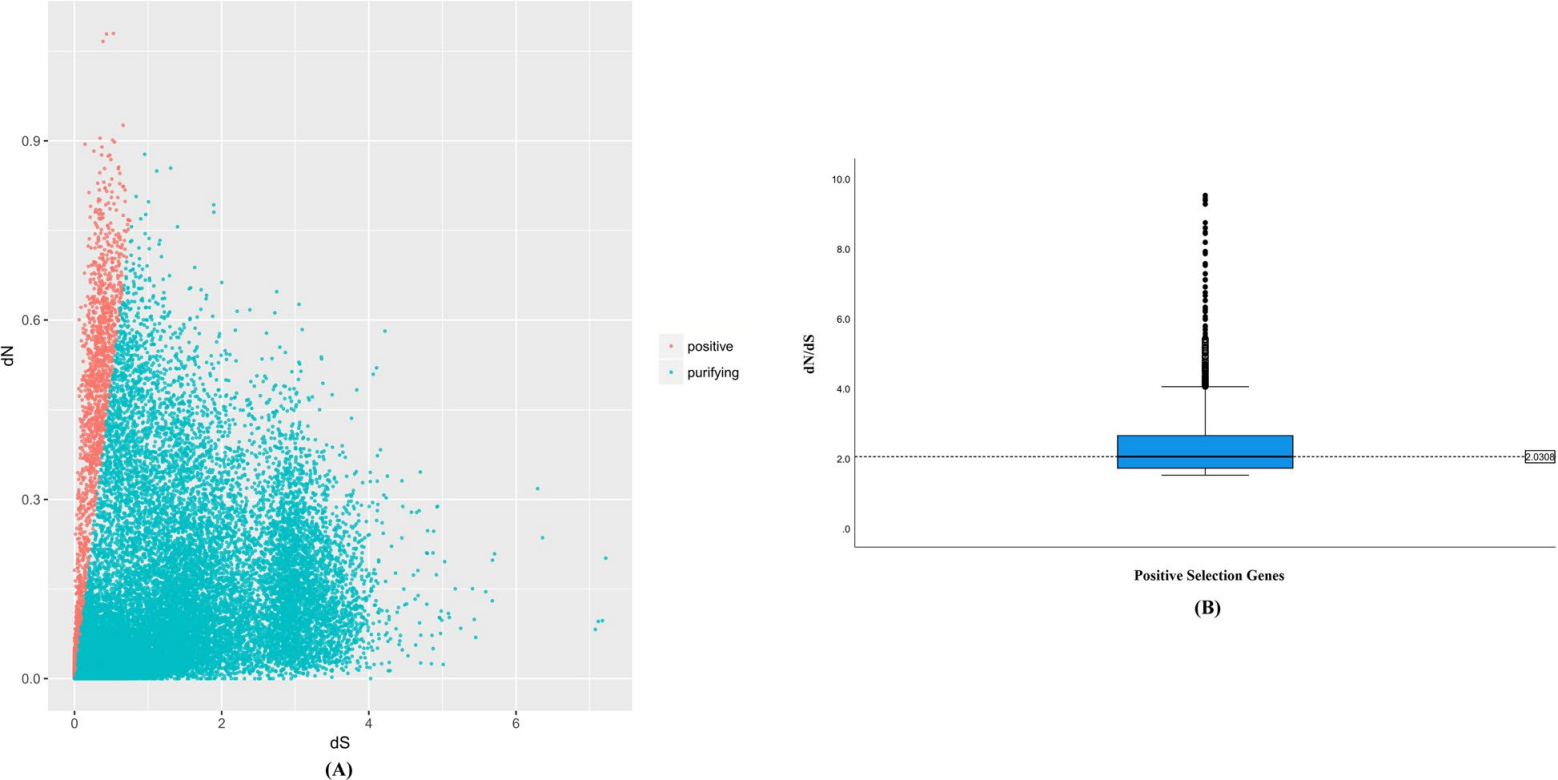
Selection pressure analysis of single copy homologous genes in *O. longilobus* and *O. taihangensis*. **A** The x-axis represents the dS (synonymous substitution rate) value, while the y-axis represents dN (non-synonymous substitution rate) value. Red dots represent single copy homologous gene pairs (Y/X > 1.5) under positive selection, and blue dots represent single copy homologous gene pairs (Y/X < 0.9) under negative selection. **B** The distributions of dN/dS ratios of the positive selection genes

**Fig. 4:**
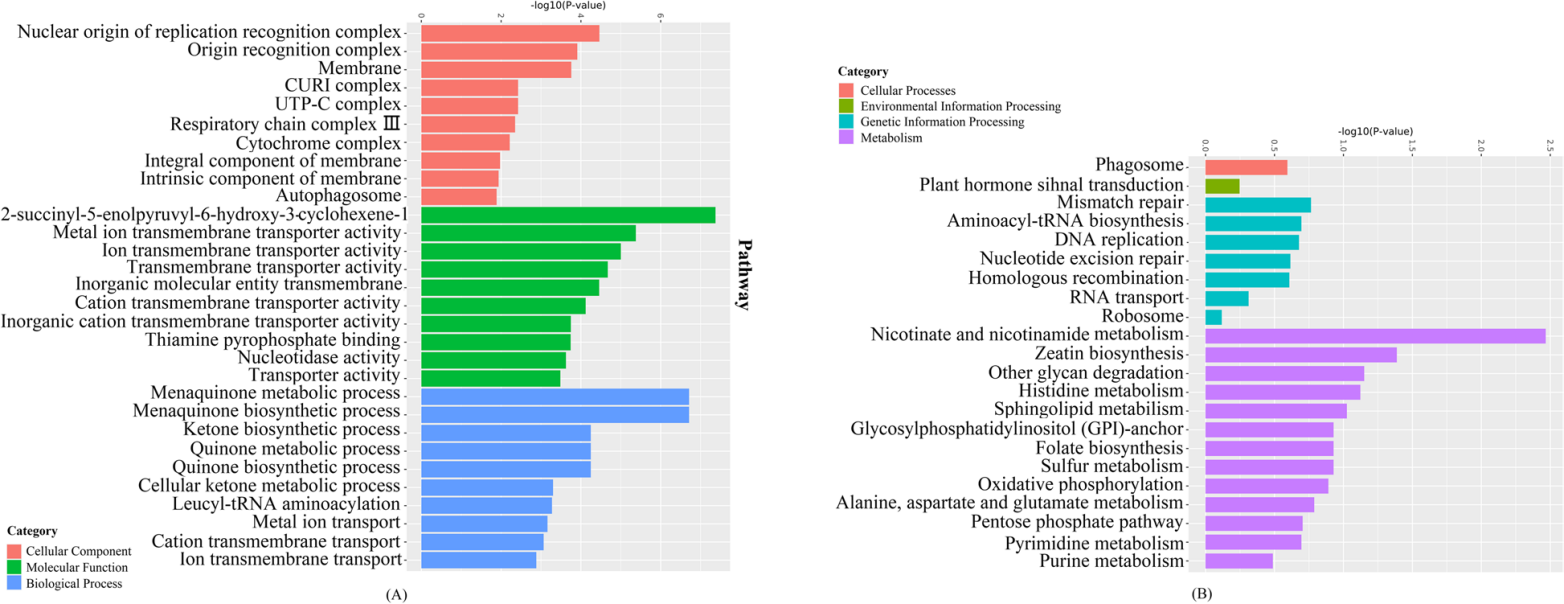
GO and KEGG enrichment analysis of positive selection genes. **A **GO analysis; **B** KEGG analysis

There were five othologous pairs with dN/dS ratios > 10 and 1203 with dN/dS ratios > 1.51 (*P *< 0.05). According the frequency distribution plots of all dN/dS ratios of two species, we took a more appropriate threshold of 1.5 for the dN/dS ratio as an indicator of positive selection [17, 22, 45] 

thus 1203 pairs with dN/dS values >1.51 were identified as undergoing positive selection (*P* < 0.05) (Fig. 3A). Among the positive selection genes, 63 were shared by the two species, and 1125 were either exclusive to *O. taihangensis* or to *O. longilobus* (Fig. 3A). All positively selection genes were shown in a boxplot distribution with a median of 2.0308 (Fig. 3B). These results suggested divergent evolutionary processes in *O. taihangensis* and *O. longilobus*. Meanwhile, we clustered the above 1203 putative orthologous pairs into three main GO categories (biological process, cellular component, and molecular function). Within the biological process category, the term ‘menaquinone metabolic process’ and ‘menaquinone biosynthetic process’ were the most dominant. Within the cellular component category, ‘nuclear origin of replication recognition complex’ and ‘origin recognition complex’ represented the major subcategories. Within the molecular function category, the main functional subcategories were ‘2-succinyl-5-enolpyruvyl-6-hydroxy-3-cyclohexene-1’ and ‘metal ion transmembrane transporter activity’ (Fig. 4A).

**Fig. 5:**
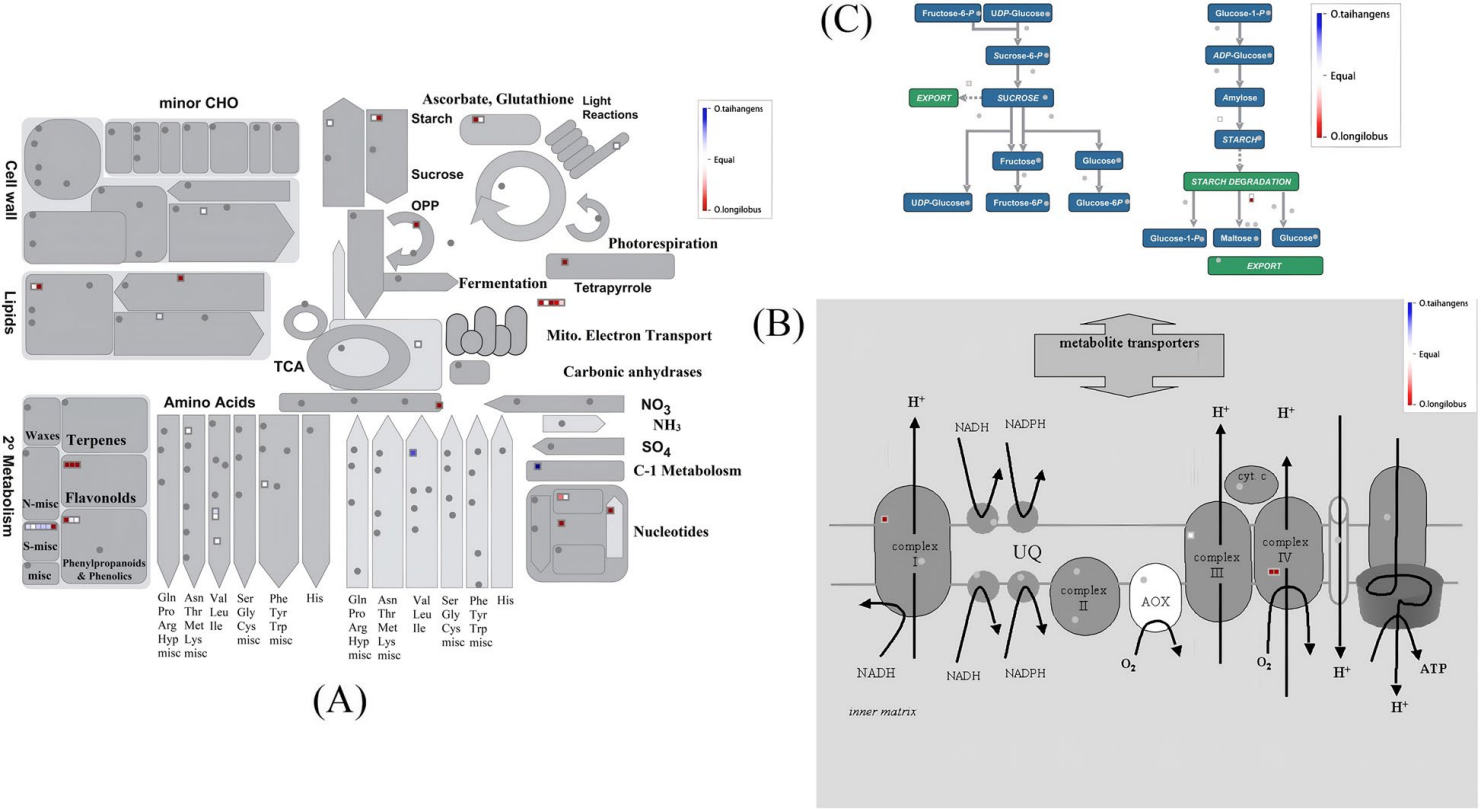
An overview of the metabolic pathways of differentially expressed positive selection genes between *O. longilobus* and *O. taihangensis.* Each inset presents a differentially expressed gene. The red lattice represents *O. longilobus*, and the blue lattice represents *O. taihangensis*. The color scale presents the fold change value of DEGs. **A** Results of mapping to Metabolism overview; **B** Mitochondrial electron transport overview; **C** Sucrose-Starch overview

Furthermore, we used KEGG pathways to investigate the biological functions of positive selection genes. Overall, the PSGs were assigned to 22 KEGG pathways. The most represented pathways were “Nicotinate and nicotinamide metabolism”, “Zeatin biosynthesis (Metabolism of terpenoids and polyketides)”, “Other glycan degradation”, “Histidine metabolism”, and “Sphingolipid metabolism” (Fig. 4B). For a comprehensive assessment of the variations/similarities in the transcriptomes of both species, PSG transcripts were plotted using the MapMan tool, which were separated by bins based on their functional ontology (Fig. 5). The functional pathway classification of 1203 PSGs mapped to the “overview of metabolism”, with 4 to lipid metabolism, 12 to secondary metabolism, 7 to amino acid metabolism, 5 to mitochondrial electron transport, 4 to nucleotide metabolism, 3 to major carbohydrate metabolism, one to the tricarboxylic acid (TCA) cycle, 2 redox ascorbate and glutathione ascorbate, 1 to oxidative PP.6-phosphogluconolactonase, one to C1-metabolism, one to cell wall metabolism, one to photosystems and one to tetrapyrrole synthesis.

Although major differences were not observed between *O. longilobus* and *O. taihangensis*, and most transcripts were shared by the two species, there were still a number of PSGs that differed. For example, the lipids pathway, major carbohydrate metabolism pathways, nucleotide metabolism pathways, tetrapyrrole metabolism pathways, mitochondrial electron transport pathways, presented higher expression levels in *O. longilobus* than in *O. taihangensis*. On the contrary, valine, leucine, and isoleucine biosynthesis in the amino acid pathway and C-1 metabolism pathways were obviously more active in *O. taihangensis* than *O. longilobus* (Fig. 5A).

We simultaneously annotated the mitochondrial electron transport / ATP synthesis pathway, nucleotide metabolism pathway and sucrose-starch pathway. An overview of mitochondrial electron transport / ATP synthesis pathway showed that the various pathways presented mainly on NADH-DH complex I and cytochrome c oxidase of the mitochondrial electron transport chains complex I and IV involved the differentially expressed PSG trinity_dn142097_c0_g1 (prohibitin 3, PHB3) (Fig. 5B). In terms of nucleotide metabolism pathway, the differentially expressed PSG trinity_dn139928_c0_g1 (inositol monophosphatase family protein) for *O. longilobus* was significantly higher than those for *O. taihangensis* (Fig. 5A). An overview of major carbohydrate metabolism pathways suggested that expression level of the PSG trinity_dn108610_c0_g1 (encodes a beta-amylase targeted to the chloroplast) involved in beta-amylase for *O. longilobus* was significantly higher than that for *O. taihangensis* (Fig. 5C).

**Fig. 6:**
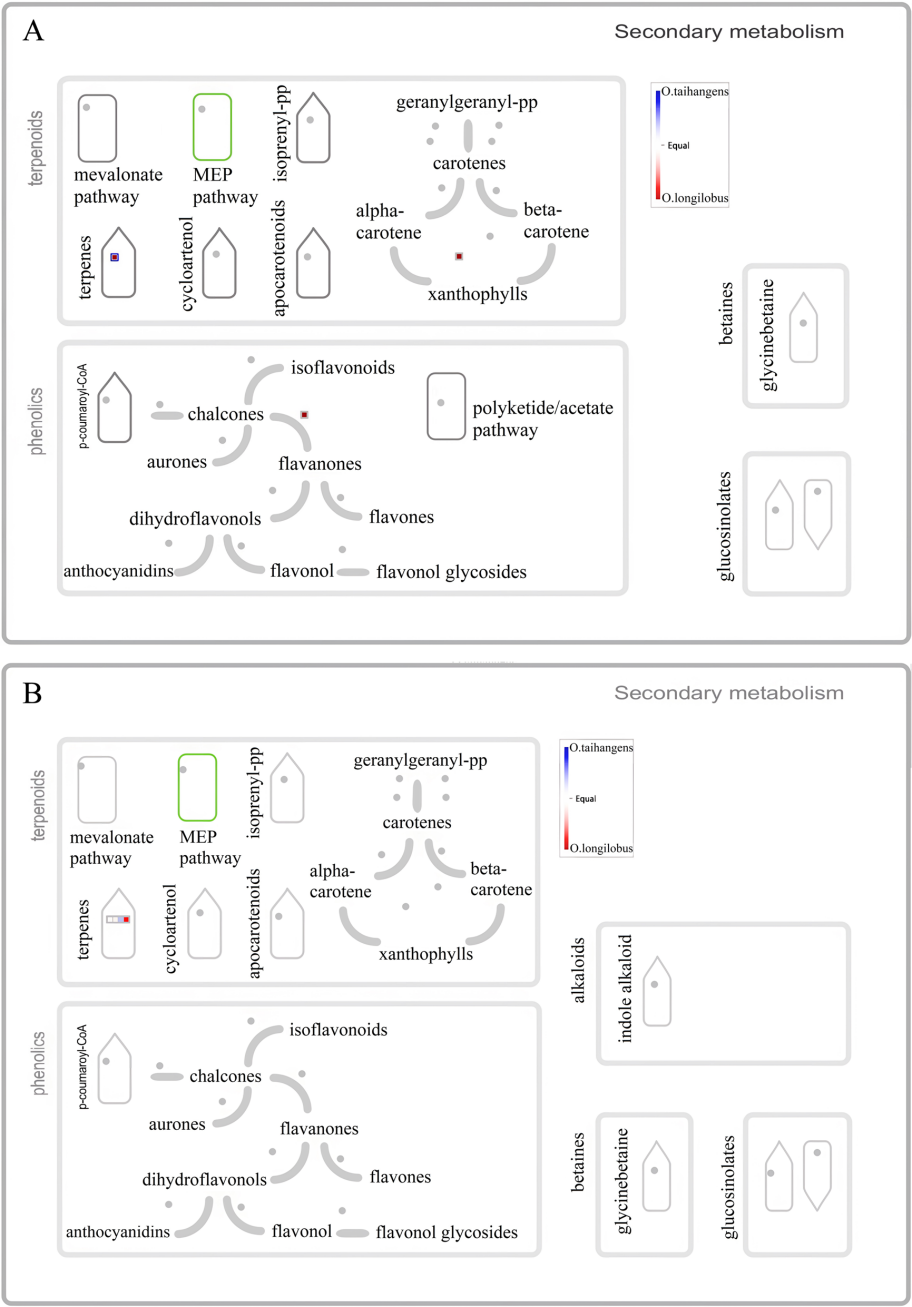
Terpenoid biosynthesis overview in secondary metabolic pathways. **A** for positive selection genes (PSGs); **B** for transcription factors (TFs). Red and blue indicate *O. longilobus* and *O. taihangensis*, respectively

**Fig. 7:**
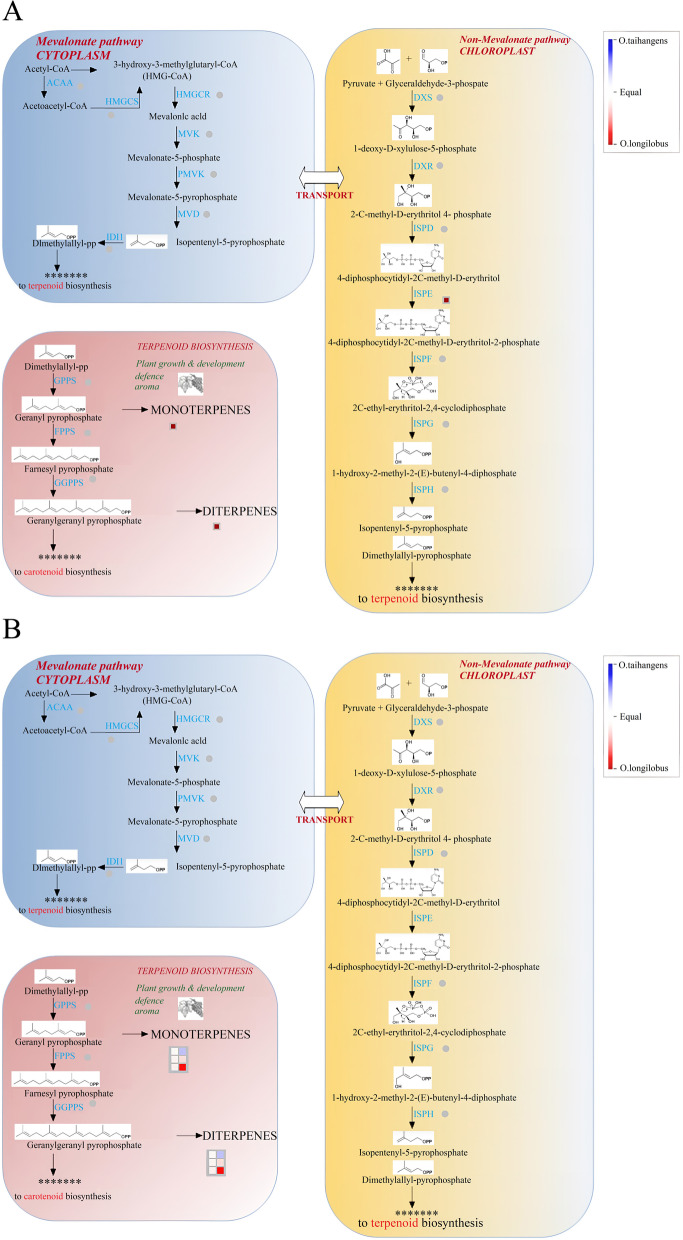
Terpene biosynthesis pathways. **A**: for positive selection genes (PSGs); **B**: for transcription factors (TFs). Red and blue indicate *O. longilobus* and *O. taihangensis*, respectively

Based on the above results, either TFs or PSGs, or both, can enrich the metabolic process pathway. We further annotated the secondary metabolite pathway of the metabolic process. The analysis of pathways enriched by MapMan revealed that terpenoid biosynthesis was simultaneously enriched at PSGs (Fig. 6A) and TFs (Fig. 6B), and only the terpene biosynthesis pathway exhibited higher expression in *O. longilobus* than *O. taihangensis*. Except for the terpene biosynthesis pathway, the PSGs trinity_dn151066_c0_g1 (annotated as chalcone isomerase) and trinity_dn141682_c0_g1 (annotated as neoxanthin biosynthesis cofactor, NXD1) were primarily identified on the flavanone biosynthesis and xanthophyll biosynthesis pathways (Fig. 6A). Furthermore, there were significantly different expression levels between the two species in these pathways. Terpenoids biosynthesis were enriched on mono-/sesquiterpene-/diterpene synthase where the expression levels of the PSG trinity_dn129788_c0_g1 (annotated as terpene synthase 14, TPS14) were higher in *O. longilobus* than *O. taihangensis* (Fig. 7A). Except for the monoterpene and diterpene biosynthesis pathways, the genes were mainly enriched on non-mevalonate pathway secondary metabolism pathways, especially ISPD (4-diphosphocytidyl-2-Cmethyl-D-erythritol synthase) and ISPE (4-diphosphocytidyl-2-C-methyl-D-erythritol kinase), where the expression levels of the PSG trinity_dn155665_c0_g2 (encodes 4-diphosphocytidyl-2-C-methyl-D-erythritol kinase, ISPE) were still higher in *O. longilobus* than *O. taihangensis* (Fig. 7A).

The terpenoid biosynthesis pathway was further analyzed by MapMan tools. The results indicated that the enriched pathway was carotenoid biosynthesis pathway with higher expression levels in *O. longilobus* than *O. taihangensis* for TFs. Moreover, this differentiation primarily occurred along the monoterpene and diterpene biosynthesis pathways. For these two pathways, the involved TFs all belonged to the bHLH family (Fig. 7B).

## Discussion

Previous studies have confirmed significant differentiation and variation between *O. longilobus* and *O. taihangensis* based on geographic distribution, morphological characteristics and DNA information [26, 27, 29–33]. Subsequently, Chai [28, 34] used transcriptome data to reveal differentially expressed genes and screened DNA markers of the two species to further prove the differentiation and variation between the two species. To uncover the environment-related molecular mechanisms underlying the differentiation and variation of the two *Opisthopapus* species, we performed a comparative analysis of genes for adaptive evolution occurring in transcriptome data, providing some clues to further elucidate the major differences under natural selection.

### Adaptive characteristics of TFs

Transcription factors (TFs) are significant key elements of various physiological and biochemical pathways in higher plants. The activation and repression of TFs can primarily regulate the expression of genes related to growth, stress responses of plants, and other developmental processes [40, 52]. Thus, TF-based gene expression regulation allows plants to respond to changes in their environments [40, 53]. Based on transcriptome data, we initially obtained 406 TFs (Table 1). The bHLH, FAR1, NAC, MYB, and WRKY family members were highest among the obtained factors. These factors were found to be significantly expressed for lipid, secondary, amino acid, and nucleotide metabolism, which constituted the main biological processes. As many TFs were involved in the amino acid metabolism pathways (Fig. 2), the proactive responses to environmental/developmental cues were explicitly depicted in *O. taihangensis*.

In particular, *O. longilobus* had a higher expression level in the terpenoid metabolic pathway than did *O. taihangensis* (Fig. 6). Furthermore, the involved TFs were all bHLH family members (Fig. 6). Basic helix-loop-helix (bHLH) transcription factors, which are one of the largest families and a large superfamily in plants, play relevant roles in a variety of developmental and evolutionarily conserved processes, including cell-fate specification, tissue differentiation, and secondary metabolites [24, 54, 55]. The bHLH factor *VvMYC1* can regulate anthocyanin and/or proanthocyanidin (PA) synthesis of the flavonoid pathway and thus regulates the biosynthesis of terpenoids [56]. Several bHLH TFs within terpenoid biosynthesis pathway have been found in *Medicago truncatula* [57], *Chenopodium quinoa* [58], *Panax notoginseng* [59], and *Panax ginseng* [60]. The differential expression of bHLH family in the two *Opisthopappus* species indicated partly adaptive differentiation and variation.

Terpenoids are one of the most common compounds in the secondary metabolites of plants, which have numerous physiological and ecological functions, such as attracting pollinated insects, regulating growth and development, resisting environmental stress, and participating in the defense of pests [61]. Meanwhile terpenoids are important components of the fragrances of flowers. For the two species of *Opisthopappus* under study, these secondary metabolites are primarily emitted by their leaves and flowers. According to the Flora Reipublicae Popularis Sinicae, the surfaces of *O. longilobus* leaves are smooth and glabrous, while *O. taihangensis* is sparsely pubescent on both leaf surfaces, which include glandular trichomes and non-glandular trichomes [62]. The terpenoids produced in *O. taihangensis* can be released through glandular trichomes which give rise to the unique aroma for communication and defense [62]. For *O. longilobus*, owing to the lack of glandular trichomes on the surfaces of its leaves, terpenoid biosynthesis genes were more highly expressed to compensate for this deficit through the use of alternative emission strategies.

The other side, many bHLH genes have been shown to respond to various forms of stress such as drought, salt, and cold stresses [52, 57, 63]. Other TFs (e.g., NAC, MYB, and WRKY) have been investigated for their capacities to improve tolerance and resistance in many plants [64–68], while participating in abiotic and biotic stress responses [18, 69, 70]. The expression of these TFs was significantly different between the two *Opisthopappus* species in overview of the metabolic pathways (Fig. 2) and secondary metabolic pathways of transcription factors (TFs) (Fig. 6B). This suggested that the differential expression of TFs (particularly bHLH family members) might play critical roles in adaptation of the two *Opisthopappus* species to heterogeneous environments.

### Adaptability of PSGs

The average dN/dS ratios across the pairs of the *Opisthopappus* species were much lower than 0.9, suggesting that purifying selection had a general influence on the evolution of most protein-coding regions (ORF) of the two species, as has been observed in other plants [17, 71]. Actually, the genes that were most under the influence of purifying selection primarily contained structural or “housekeeping” genes, for example tyrosyl-tRNA synthetase. As these genes are involved in processes that are crucial for organisms, purifying selection can eliminate deleterious, nonsynonymous mutations [17, 72, 73]. Among the 38,986 orthologs shared between lineages, 1203 gene pairs significantly exhibited dN/dS ratios > 1.5 (*P* < 0.05) (Fig. 3A, 3B). These genes were involved in several biological functions (e.g., metabolic processes) and may constitute candidates that are under the effect of positive selection; thus, potentially associated with species divergence.

Adaptive divergence at molecular level may be reflected by an increased rate of non-synonymous changes within genes involved in adaptation [74]. Analyses of the PSGs identified from *O. taihangensis* and *O. longilobus* yielded significantly different landscapes of biological processes, cellular component and molecular function (Fig. 4). *O. taihangensis* and *O. longilobus* might experience divergent adaptation during their evolution, and genes related to adaptation was under rapid evolution and/or have signs of positive selection. These findings implicated that *Opisthopappus* were experienced the ongoing accelerated evolution under different environment. Thus, more than 1000 genes under positive selection between the two *Opisthopappus* species might have played a role in shaping the divergence of this genus, given that *O. longilobus* and *O. taihangensis* are exposed to sub-humid warm temperate, and temperate continental monsoon climates, respectively.

The comparative transcriptome analysis [28] indicated that 3,410 differentially expressed genes were mainly involved in lipid, carbohydrate and amino acid metabolism, xenobiotics biodegradation and metabolism. In this study, KEGG enrichment and MapMan analyses suggested that significant GO terms of 1203 differentially expressed positive selection genes mainly related to metabolic regulation (Fig. 4B) were important among the *Opisthopappus* species (Fig. 5). Secondary metabolism was also significantly represented for *O. longilobus* and *O. taihangensis* in our PSG analysis (Fig. 6).

Beta-amylase, a member of family 14 of glycosyl hydrolases [75], can hydrolyzes the α-1,4-glucosidic linkages in starch, removing successive maltose units from the non-reducing ends of the chains [76]. Starch is the main form of carbon storage in higher plants and it accumulates in different organs, which can be consumed by the cell in which they are produced, transported to nonphotosynthetic sink tissues, or stored for later use. According to studies using transgenic and mutant plants, beta-amylase seems to be important for normal degradation of the transient starch accumulated in chloroplasts [76]. Meanwhile, beta-amylases possibly facilitating the rapid starch degradation under heat and drought [77]. In this study, we found that the PSG expression levels involved in beta-amylase for *O. longilobus* was significantly higher than those for *O. taihangensis* (Fig. 5A). Starch degradation as a major response in conditions prohibiting photosynthesis or prolonged drought [77], therefore, *O. longilobus* seems better adapted to weak light or drought conditions than *O. taihangensis*.

Beta-amylase can convert starch into maltose and glucose as the predominant form in which sugars are translocated in plants presented in Fig. 5C. Consistent with this notion, *O. longilobus* exhibited higher levels of cellular glucose and fructose than did *O. taihangensis*. Collectively, these data signified that the higher expression levels of beta-amylase genes in *O. longilobus* might accelerate its cell growth processes (in contrast to *O. taihangensis*), which have important roles in the evolution of the two species. Ultimately, glycan degradation resulted in the accumulation of sucrose, which is critical for the protection of plants against xenobiotic and oxidative stresses [18], and which might facilitate the enhanced survival of *O. longilobus* for relatively more drought habitats over than *O. taihangensis*.

The electron transport chain (ETC, respiratory chain) is a series of protein complexes that transfer electrons from electron donors to electron acceptors via redox reactions (both reduction and oxidation occurring simultaneously) and couples this electron transfer with the transfer of protons (H + ions) across a membrane. The electron transport chain is built up of peptides, enzymes, and other molecules. In this study, the expression levels of relative PSGs involved in complex I (NADH ubiquinone oxireductase) and complex IV (cytochrome c oxidase, EC 1.9.3.1) for *O. longilobus* was higher than those for *O. taihangensis* (Fig. 5B). The ETC sustains the major mitochondrial function of ATP generation, in relation to the metabolic dynamics of tricarboxylic acids (TCAs), acetyl-CoA, ADP, oxidized (NAD +) or reduced (NADH) β-nicotinamide adenine dinucleotide, oxidized (FAD) or reduced (FADH2) flavin adenine dinucleotide [78]. Thus, we concluded that the changes in ETC can affect nicotinate and nicotinamide metabolism, glycan degradation metabolism and transmembrane transport activity, which was supported by the KEGG and MapMan results (Figs. 4 and 5). The various expression levels of relative PSGs between these two species were also regarded as different adaption signals to habitats.

Additionally, the responses to sulfur between *O. longilobus* and *O. taihangensis* were significantly different, which suggested that the two species possessed different stress tolerances for S in response to heterogeneous environments.

### Adaptive function of Terpenoids

*O. taihangensis* and *O. longilobus* secrete chemicals and exude aromas to protect against biological and abiotic stresses [62]. These chemicals are primarily secondary metabolites, including polyphenols, alkaloids, and terpenoids. This scenario, particularly in terms of terpenoid biosynthesis, was unambiguously observed in the present study as a difference between the two species TFs and PSGs (Fig. 6). The terpenoid pathway is intricately regulated by endogenous and environmental factors that enable spatially and temporally controlled metabolite production [79–81]. Terpenes comprise the largest and the most diverse class of natural products, often play important roles in plant defenses by attracting the enemies or predators of herbivores and repelling herbivorous insect feeders [82].

Isopentenyl pyrophosphate (IPP) and dimethylallyl pyrophosphate (DMAPP) are the main intermediate compounds in the terpenoid pathway for terpene biosynthesis [20, 83]. Their precursor molecules (C5) are generated via the process of isoprenogenesis [84, 85]. In plants, isoprenogenesis occurs through two discrete biosynthetic pathways: the mevalonic acid (MVA) pathway in the cytosol, and the 2-C-methyl-D-erythritol 4-phosphate/1-deoxyD-xylulose 5-phosphate (MEP/DOXP) pathway within plastids. In the MEP pathways, the C5 units are catalyzed step by step via a series of enzymes to synthesize IPP. The 4-diphosphocytidyl-2C methyl-D-erythritol synthase (ISPD/CMS) and 4-diphosphocytidyl-2C-methyl-D-erythritol kinase (ISPE/CMK) catalyze the synthesis of 4-diphosphocytidyl-2C-methyl-D-erythritol (CDP-ME) and 4-diphosphocytidyl-2C-methyl-D-erythritol 2-phosphate (CDP-ME2P), respectively. CDP-ME and CDP-ME2P are the intermediate compounds of IPP biosynthesis.

IPP or DMAPP may be linked through head to tail condensation reactions to generate terpenes of different classes (e.g., mono, di, and triterpenes), which are catalyzed by geranyl diphosphate synthase (GPPS) to synthesize geranyl pyrophosphate (GPP). The GPP results in farnesyl pyrophosphate (FPP) and geranylgeranyl diphosphate (GGPP) via farnesyl pyrophosphate synthase (FPPS) and geranylgeranyl diphosphate synthase (GGPPS) enzymes, respectively. GPP and GGPP are substrates for monoterpene and diterpene biosynthesis in the terpene pathway [86–88]. Monoterpenes and diterpenes can impart the distinct flavors and aromas of plants [20, 89]. These terpenes are produced in plants as secondary bioactive metabolites, often for ecological adjustment and protection from microbial pathogens, fungi, pests, and predation [90]. An overview of terpenoid metabolism of the two species revealed that the putative PSG displayed different levels of expression for IPP biosynthesis, particularly in the synthesis of CDP-MK and CDP-ME2K (Fig. 7).

Meanwhile, both the PSGs and TFs involved in the downstream steps of the MEP pathway exhibited a relatively higher expression in contrast to the two species under study, which displayed different expression levels for monoterpene and diterpene biosynthesis (Fig. 7). These results indicated variable adaptability in the responses of the two species to different environments. During the evolutionary process, the genes (such as trinity_dn129788_c0_g1, annotated as terpene synthase 14, TPS14) involved in terpenoid biosynthesis pathway (in particular, monoterpene and diterpene biosynthesis), begun to diverge from their expression levels under different long-term environment stresses. Simultaneously, several bHLH TFs involved this metabolic process to further regulate the expression of these positive selection genes. Ultimately, the adaptive phenotypic characteristics occurred in *O. longilobus* and *O. taihangensis*, such as the presence of trichomes or not, and different fragrances.

## Conclusion

This work presented a comparative genome-wide transcriptome analysis between two *Opisthopappus* species, toward the identification of biological processes and functional genes that facilitate differentiation and adaption of these two species during evolutionary processes. Under natural selection, *O. taihangensis* gradually modified its gene expression model (such as mitochondrial electron transport and starch degradation) to adapt to its surroundings, which differed from *O. longilobus*. The screened several PSGs and TFs involved in the terpenoid biosynthesis of monoterpenoids and diterpenoids, and represented adaptive signal responses to heterogeneous environments. These results will not only shed light on how differentiations between the two *Opisthopappus* species occur, but also open the door to an increased understanding of how plants thrive in mountains environments. Additionally, our research lays a theoretical foundation for the further investigation of the molecular mechanisms of specific characteristics that have important academic and application value for other Asteraceae species.

## Data Availability

Data deposited via the Genebank: accession SUB4002238: PRJNA471103.
